# Improved antiretroviral treatment outcome in a rural African setting is associated with cART initiation at higher CD4 cell counts and better general health condition

**DOI:** 10.1186/1471-2334-11-98

**Published:** 2011-04-19

**Authors:** Erik Mossdorf, Marcel Stoeckle, Emmanuel G Mwaigomole, Evarist Chiweka, Patience L Kibatala, Eveline Geubbels, Honoraty Urassa, Salim Abdulla, Luigia Elzi, Marcel Tanner, Hansjakob Furrer, Christoph Hatz, Manuel Battegay

**Affiliations:** 1 St. Francis Designated District Hospital, Ifakara, United Republic of Tanzania; 2 Ifakara Health Institute, Ifakara, United Republic of Tanzania; 3 Ifakara Health Institute, Dar es Salaam, United Republic of Tanzania; 4 Swiss Tropical and Public Health Institute, University Basel, Basel, Switzerland; 5 Division of Infectious Diseases & Hospital Epidemiology, University Hospital Basel, Basel, Switzerland; 6 Division of Infectious Diseases, University Hospital and University of Berne, Berne, Switzerland

**Keywords:** HIV-1, antiretroviral therapy, treatment outcome, rural, Tanzania

## Abstract

**Background:**

Data on combination antiretroviral therapy (cART) in remote rural African regions is increasing.

**Methods:**

We assessed prospectively initial cART in HIV-infected adults treated from 2005 to 2008 at St. Francis Designated District Hospital, Ifakara, Tanzania. Adherence was assisted by personal adherence supporters. We estimated risk factors of death or loss to follow-up by Cox regression during the first 12 months of cART.

**Results:**

Overall, 1,463 individuals initiated cART, which was nevirapine-based in 84.6%. The median age was 40 years (IQR 34-47), 35.4% were males, 7.6% had proven tuberculosis. Median CD4 cell count was 131 cells/μl and 24.8% had WHO stage 4. Median CD4 cell count increased by 61 and 130 cells/μl after 6 and 12 months, respectively. 215 (14.7%) patients modified their treatment, mostly due to toxicity (56%), in particular polyneuropathy and anemia. Overall, 129 patients died (8.8%) and 189 (12.9%) were lost to follow-up. In a multivariate analysis, low CD4 cells at starting cART were associated with poorer survival and loss to follow-up (HR 1.77, 95% CI 1.15-2.75, p = 0.009; for CD4 <50 compared to >100 cells/μl). Higher weight was strongly associated with better survival (HR 0.63, 95% CI 0.51-0.76, p < 0.001 per 10 kg increase).

**Conclusions:**

cART initiation at higher CD4 cell counts and better general health condition reduces HIV related mortality in a rural African setting. Efforts must be made to promote earlier HIV diagnosis to start cART timely. More research is needed to evaluate effective strategies to follow cART at a peripheral level with limited technical possibilities.

## Background

WHO launched the ambitious "3 by 5" strategy in 2003 following the dramatically improved prognosis of HIV-infected patients receiving combination antiretroviral therapies (cART) in industrialized countries [1,2]. This public health approach aimed to introduce cART at large scale for resource-constrained countries [3] carrying most of the HIV attributable disease burden [4]. WHO's policy of "universal access" followed in 2007 [5] based on publications reflecting comparable clinical and immunological outcomes under cART from different resource-limited countries [6-17]. The number of people receiving cART in low- and middle-income countries has increased 13-fold since 2004 and by the end of 2009, an estimated 5.2 million people were receiving cART, which represent 36% of those who need treatment [4].

In 2007, the HIV-prevalence in Tanzania was estimated to be 5.7% with about 1,867,918 HIV infected Tanzanian children and adults [18,19]. The HIV incidence slowed to about 3.4/1000 person-years between 2004 and 2008 [4]. In response to the HIV epidemic, the Government of Tanzania launched the "National HIV/AIDS Care and Treatment Plan 2003 - 2008" - an initiative to prevent HIV/AIDS and to provide treatment and care for patients living with HIV/AIDS [20]. By the end of 2007, 127,895 HIV infected persons received cART with an increase to 154,468 only one year later [4,19]. As in other countries, the Tanzanian treatment plan was initiated first in large cities at university and/or referral hospitals. However, a significant part of HIV-infected Tanzanians live in rural regions [21,22] and cART evaluations from rural care and treatment centres in sub-Saharan Africa, and in particular Tanzania, are still scarce [13,23-27].

We aimed to assess the clinical and immunological response to cART in a rural treatment centre in Tanzania emphasizing immunological recovery and risk factors of death or loss to follow-up during the first year after starting cART.

## Methods

### Study design and setting

We analyzed data of a local prospective cohort study of HIV-infected individuals at the Care and Treatment Centre of St. Francis Designated District Hospital (SFDDH) in Ifakara, Tanzania. All HIV-infected adults initiating cART between 1^st ^January 2005 and 20^th ^December 2008 at the SFDDH were included in this study.

The SFDDH is the most important health care facility in the rural Kilombero and Ulanga District of the Morogoro Region in Southern Tanzania, providing treatment and care for a population of about 600,000 inhabitants and an estimated 30,000 patients living with HIV/AIDS [5]. Established in 2004, the Chronic Disease Clinic at St. Francis Designated District Hospital was the first rural clinic accredited to be a Care and Treatment Centre of the National AIDS Control Programme (NACP) in the whole of Tanzania [28]. By December 2008, the Care and Treatment Centre at SFDDH had enrolled 3,440 patients living with HIV/AIDS. Of these, 2,445 were followed-up on a permanent basis, and 1,491 treated with cART. Each patient taking cART had a personal adherence supporter [28]. In addition, all patients presenting with tuberculosis were tested for HIV, enabling thereby early diagnosis and follow-up of HIV/tuberculosis co-infected patients. After initiation of cART and clinical stabilization, patients were referred to a Refilling Centre closer to their living place facilitating adherence, and returned to the Care and Treatment Centre only on a three-monthly basis.

### Outcome measures and cART

In the survival analysis, we included all HIV-infected patients who died from all causes within the first year after starting cART. We also considered all patients lost to follow-up as failures, since a meta-analysis of Brinkhof et al. [29] showed that in rural sub-Saharan African cohorts up to 20 to 69% of patients lost to follow-up that could be retraced had died.

All patients were treated according to NACP guidelines [30] which are based on the 2002 WHO recommendations and the 2007 revision of the WHO guidelines [31]. Individuals were eligible for cART if they: (a) had a WHO stage 4 disease, (b) had proven tuberculosis and a CD4 cell count between 200 and 350 cells/μl, (c) had a CD4 cell count below <200 cells/μl or (d) were pregnant irrespective of CD4 cell count. In addition, every patient with a WHO stage 3 and more or a CD4 cell count <200 cells/μl received prophylaxis with co-trimoxazole 400/80 mg/day.

Patients were followed up every month until resolution of clinical symptoms of an AIDS defining illness and to the point of achieving two consecutively increasing CD4 cell counts under cART. Thereafter, appointments were scheduled on a three-monthly basis. At each visit, signs of tuberculosis were actively investigated. Laboratory controls included a blood cell count and chemistry two weeks after initiation of cART and a CD4 cell count after 3 months and every 6 months thereafter. Measurement of HIV-RNA was not available for the present study. cART switches due to toxicity or other reasons were performed in line with the NACP [30]. The same applied for treatment failures defined as a new or recurrent WHO stage 4 condition, decrease of CD4 cell count to pre-therapy baseline (or below), a 50% fall from the on-treatment peak value or persistent CD4 levels below 100 cells/μl.

### Adherence support

Each patient receiving cART designated a personal adherence supporter - mostly a family member or any other trustworthy person living in the vicinity of the patient. The supporters received counseling about cART and side effects, provided assistance in taking cART throughout the study period and accompanied the patient at cART initiation and from then on request at follow-up visit.

Patients with cART who missed visits were contacted or traced by home based care volunteers where possible.

### Data collection and definitions

Data was collected prospectively using standardized record forms completed at each follow-up visit including information on the clinical course, occurrence of tuberculosis, WHO stage, adherence, laboratory results and treatment history. Data was entered into the NACP and the Kilombero and Ulanga Antiretroviral Cohort (KIULARCO) databases. Quality control was performed by double data entering and cross-checking procedures.

Staging of HIV disease followed the case and stage definition according to WHO guidelines [32]. Tuberculosis was diagnosed and treated following WHO guidelines within the framework of the National Tuberculosis and Leprosy Control Programme [33].

cART defined as a combination of at least 3 antiretroviral drugs was prescribed following the recommendations of the NACP [30]. The first-line regime consisted of a fixed dose combination of nevirapine 200 mg plus lamivudine 150 mg and stavudine 30 mg (individuals > 60 kg 40 mg till end of December 2006) twice daily. Similar immunological and virological outcomes were found between the two-stavudine doses in patients weighing more than 60 kg in an African operational cohort at 6 months [34]. Alternatives were 1 tablet of nevirapine 200 mg plus a fixed dose combination of lamivudine 150 mg and zidovudine 250 mg twice daily, or 1 tablet of efavirenz 600 mg once daily either with lamivudine 150 mg plus zidovudine 250 mg as a fixed dose combination or lamivudine 150 mg plus stavudine 30 mg (individuals > 60 kg 40 mg till end of December 2006) twice daily. The second-line treatment was a fixed dose combination of 2 pills of lopinavir/ritonavir 200/50 mg plus 1 tablet of abacavir 300 mg twice daily plus 4 tablets of didanosine 100 mg once daily. Treatment modification was defined as any change of one or more cART components excluding dosage adjustments. The main reason for treatment modification was classified as failure (clinical, immunological), toxicity (nausea/vomiting, rash, peripheral neuropathy, hepatitis, anemia, CNS adverse events), interactions (start of TB treatment) or others. Anemia was defined as haemoglobin < 12 g/dl and was seen as a contraindication for prescribing zidovudine. Hepatitis was diagnosed if transaminases increased > 5 times the upper norm.

Loss to follow-up was defined as not showing up on two consecutive visits, i.e. for a period of 6 months.

### Statistical analysis

Basic socio-demographic characteristics, co-infection with tuberculosis, HIV clinical stage, CD4 cell count, and antiretroviral treatment were compared using the Chi-square test or Fisher's exact test for categorical variables, and the Mann-Whitney test for continuous variables. Cox proportional hazard models were used to investigate factors associated with survival and loss to follow-up after starting cART. All patients were censored at 12 months after starting cART if no death or loss to follow-up had occurred. Patients who were lost to follow-up were considered as failures and censored at the last visit date. The proportional hazards assumption was checked using the Schoenfeld test. Logistic regression was used to explore risk factors of being lost to follow-up during the first year of cART and as well as predictors of treatment modification. Multivariate models were built using a forward stepwise approach, adding each factor significant at the level of 0.1 and other factors defined in the literature as prognostic in the model one by one. The final model was examined for interaction using the likelihood ratio test.

All analyses were performed using STATA™ software version 9.2 for Windows (StataCorp, College Station, Texas).

### Ethical approval

The research and ethical clearance was obtained from the Medical Research Coordination Board of the National Institute for Medical Research, Tanzania, through the Tanzania Commission for Science & Technology and from the Ethical Review Board of the Cantons Baselstadt and Baselland, Switzerland. Written informed consent was obtained from all participants enrolled in the Kilombero and Ulanga Antiretroviral Cohort (KIULARCO).

## Results

Overall, 1,463 treatment-naïve, HIV-infected adults initiated cART between 1^st ^January 2005 and 20^th ^December 2008. The median age was 40 years (interquartile range [IQR] 34-47), 35.4% were males, and 7.6% had confirmed tuberculosis. 2.6% of the female individuals were pregnant. The median CD4 cell count was 131 cells/μl (IQR 54-234) and 24.3% had a WHO clinical stage 4. Patients co-infected with tuberculosis tended to start cART at higher CD4 cell counts than patients without tuberculosis (142 versus 117 cells/μl, p = 0.060). Antiretroviral therapy consisted of a nevirapine-based regimen in 83.1% of individuals. 75% of all patients were started on nevirapine/lamivudine/stavudine, 8.1% on nevirapine/lamivudine/zidovudine, 13.3% on efavirenz/lamivudine/zidovudine, 1.8% on efavirenz/lamivudine/stavudine, and 1.8% on other drug combinations.

General characteristics of the study population according to the outcome at 12 months are listed in Table 1.

**Table 1:** General characteristics of the adult study population (n = 1463) according to the outcome at 12 months after starting combination antiretroviral therapy

Characteristics at 12 months		All patients N = 1463		Death or lost to follow-up N = 318		Alive and on cART N = 1145		p-value dead vs. alive
		n	%	n	%	n	%	
Median age, IQR		40	34-47	40	33-48	40	34-47	0.913
Males		518	35.4	130	40.9	388	33.9	**0.021**
Marital status	Single	213	14.6	24	7.6	189	16.5	**<0.001**
	Married	390	26.7	48	15.1	342	29.9	
	Divorced or Widowed	277	18.9	37	11.6	240	21.0	
	No information	583	39.8	209	65.7	374	32.7	
Median weight, IQR		50	43-56	45	39-52	51	44-57	**<0.001**
Median hemoglobin, IQR		9.5	8-11	8.8	7.4-10.7	9.8	8.2-11.1	**<0.001**
WHO stage	I	162	11.1	11	3.5	151	13.2	**<0.001**
	II	155	10.6	7	2.2	148	12.9	
	III	279	19.0	31	9.8	248	21.7	
	IV	197	13.5	40	12.6	157	13.7	
	No information	670	45.8	229	72.0	441	38.5	
Median CD4 cell count, IQR		131	54-234	80	27-156	130	60-205	**<0.001**
Tuberculosis	Proven	111	7.6	19	6.0	92	8.0	**<0.001**
	Suspected	538	36.8	62	19.5	476	41.6	
cART	Nevirapine-based	890	60.8	130	40.9	760	66.4	**<0.001**
	Efavirenz-based	162	11.1	23	7.2	139	12.1	
	No information	411	28.1	165	51.9	246	21.5	
Distance to clinic	<10 km	710	48.5	143	45.0	567	49.5	0.561
	10-50 km	271	18.5	66	20.7	205	17.9	
	50-100 km	276	18.9	59	18.5	217	19.0	
	>100 km	134	9.2	33	10.4	101	8.8	
	No information	72	4.9	17	5.4	55	4.8	

### Response to cART

The median CD4 cell count increase was 61 cells/μl (IQR 10-167) and 130 cells/μl (IQR 43-222) after 6 and 12 months, respectively. In particular, at 6 months and 12 months 49.9% and 70.1% of patients with available data had CD4 cell counts > 200 cells/μl, respectively, compared with only 23.6% at starting cART. During the follow-up, the median increase in body weight was 3 kg (IQR 0-8), and 115 out of 181 patients (63.5%) with available data experienced a change of activity from either bedridden or ambulant to working. Overall, 129 patients (8.8%) died and 189 (12.9%) were lost to follow-up during the first year of cART.

### Risk factors of poor survival at 12 months after starting cART

**Figure 1:**
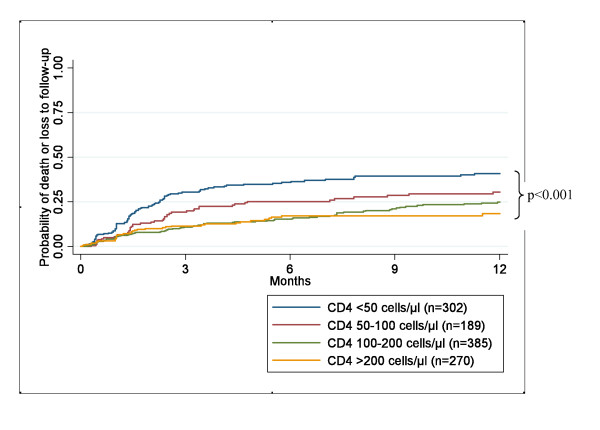
**Time to death or loss to follow-up during the first year after starting cART according to the baseline CD4 cell count cells/μl**.

In a multivariate model adjusted for age, gender, body weight, CD4 cell count, the presence of tuberculosis and antiretroviral regimen, risk factors for poor survival were lower CD4 cell counts at starting cART (HR 1.77, 95% CI 1.157-2.72, p = 0.009 for CD4 cell count <50 compared with >100 cells/μl) (Table 2 and Figure 1). In contrast, higher body weight was strongly associated with better survival (HR 0.63, 95% CI 0.51-0.76, p < 0.001 per 10 kg increase) (Table 2). Risk factors for poor survival and loss to follow-up during the first year of cART are listed in Table 2.

**Table 2:** Risk factors for poor survival and loss to follow-up during the first year of cART, hazard ratios (HR), univariate and multivariate analysis

Characteristics		Univariate analysis			**Multivariate analysis**^**§**^		
		HR	95% CI	p-value	HR#	95% CI	p-value
Age, per 10 years older		0.98	0.87-1.09	0.669	0.97	0.80-1.17	0.760
							
Female		0.77	0.62-0.97	0.025	0.77	0.52-1.15	0.201
							
Weight, per 10 kg increase		0.61	0.54-0.69	<0.001	0.63	0.51-0.76	**<0.001**
							
Tuberculosis	Absent	1*	-	-	1*	-	-
	Suspected	0.49	0.37-0.68	<0.001	0.63	0.44-1.10	0.082
	Confirmed	0.73	0.45-1.16	0.180	1.28	0.70-2.33	0.421
							
CD4 cell count	>100	1*	-	-	1*	-	-
	50-100	1.50	1.07-2.11	0.018	1.37	0.82-2.29	0.230
	<50	2.31	1.77-3.04	<0.001	1.77	1.15-2.72	**0.009**
							
cART	Nevirapine-based	1*	-	-	1*	-	-
	Efavirenz-based	1.13	0.71-1.79	0.607	0.91	0.54-1.52	0.712

# adjusted for all variables listed in the table

* reference category

§ Global test of proportional hazards assumption (Schoenfeld): p = 0.248

### Treatment modification

During the follow-up, 215 individuals (14.7%) changed their antiretroviral treatment after a median time of 133 days (IQR 58-371). The most frequent reason for modifying cART was toxicity to cART (56.2%), i.e. peripheral polyneuropathy in 26.8%, concern of potential drug-drug interactions related to antituberculous therapy in 23.2%, anemia in 13.9% of patients, and treatment failure in 6.7%. Among patients with treatment change due to toxicity, the use of stavudine was clearly associated with an increased risk of polyneuropathy (OR 28.7, 95% CI 3.85-214.34, p = 0.001), whereas all 26 patients who changed their treatment due to anemia were initiated on a zidovudine-containing regimen. Only one woman modified her initial, efavirenz-containing antiretroviral therapy due to pregnancy.

The immunological recovery at 12 months did not differ between patients who changed their treatment and those who did not (144 cells/μl [IQR 62-230] versus 122 cells/μl ([IQR 33-222], p = 0.289). However, patients who changed cART showed a higher median increase in body weight than those who remained on their first regimen (5 kg [IQR 0.2-10] versus 3 kg [IQR 0-7], p = 0.003). No difference in the mortality rate was noted according to whether patients had changed their treatment (7.9% versus 9.8%, p = 0.388).

In a multivariate model adjusted for age, sex, CD4 cell count, body weight, presence of tuberculosis, and cART, an antiretroviral regimen containing efavirenz and zidovudine combined with lamivudine (odds ratio (OR) 1.80, 95% CI 1.11-2.90, p = 0.016, compared to a nevirapine based regimen) was the only risk factor of treatment modification within the first year of cART.

### Loss to follow-up

During the study period, 189 (12.9%) patients were lost to follow-up. In a multivariate model adjusted for age, sex, marital status, distance between living place and the clinic, confirmed tuberculosis and CD4 cell count, independent risk factors of loss to follow-up were a CD4 cell count below 100 cells/μl at starting cART (OR 2.55, 95% CI 1.45-4.50, p = 0.001) and living more than 100 km away from the clinic (OR 3.02, 95% CI 1.40-6.56, p = 0.005).

### Death

Overall, 129 patients (8.8%) died during the first year of antiretroviral therapy. 56 (75.7%) of all patients with a known cause of death died of an AIDS-defining event (tuberculosis in 30.4%, bacterial pneumonia in 19.6%, *pneumocystis jirovecii *pneumonia in 14.3%, waisting syndrome in 10.7%, Kaposi sarcoma in 10.7%, cerebral toxoplasmosis in 7.1%, cervical/anal cancer in 3.6% and candida osophagitis in 3.6%). 18 individuals died of other causes (gastroenteritis in 5, cardiovascular disease in 4, severe anemia in 3, malaria in 2, peritonitis in 2, rectum carcinoma in 1 and road accident in 1 patient). The cause of death remained unknown in 55 patients.

Time to death or loss to follow-up during the first year after starting cART according to the baseline CD4 cell count cells/μl is shown in Figure 1.

## Discussion

In this prospective cohort study including 1,463 HIV-infected treatment-naïve adults who started cART between 2005 and 2008 assisted by a personal adherence supporter, improved survival was associated with higher initial CD4 cell counts and better general health condition. One out of 7 individuals changed their cART, mostly due to adverse side effects such as peripheral polyneuropathy and anemia. The immunological recovery was comparable to other studies, although importantly a considerable number of HIV-infected patients died or were lost to follow-up during the first year of cART possibly influencing this latter analysis by a selection bias.

### Study population and treatment response

The age-sex distribution of this cohort was comparable to most studies from sub-Saharan Africa with about one third being male [12,14,23,35-39]. The median CD4 cell count at cART initiation, i.e. 131 cells/μl, was slightly higher than in some settings [8,14,23,35-38]. Two cohorts [12,40] from Zambia and Rwanda and a recent systematic review in sub-Saharan Africa [41] showed a high variation of CD4 cell counts at baseline with mostly comparable CD4 cell counts at baseline. Patients co-infected with tuberculosis tended to start cART at higher CD4 cell counts, possibly reflecting earlier diagnosis of HIV in symptomatic patients. The median CD4 cell increase of 130 cells/μl after 12 months of cART was comparable to the immunological recovery reported in the large cART-LINC Collaboration multinational analysis and the Rwandan National Programme [36,37,40], but was lower than in smaller cohorts [12,14,38,39,42-44]. Importantly, CD4 cells surpassed the 200 CD4 cell level in most individuals in our cohort.

### Treatment change and toxicity

Due to differences in study modeling, in the observation period and in assessing the reason for switching, comparison of the rate of treatment change was prone to misinterpretation in the different studies evaluated [8,14,35,43,45,46]. This was additionally enhanced by the fact that toxicity due to stavudine accumulated over time whereas toxicity to nevirapine, efavirenz, lamivudine and zidovudine occurred earlier in the course of cART. The range of cART switches lay between 8%-21.9% in sub-Saharan Africa [8,14,35,43,45,46]. However, we found a higher rate of polyneuropathy despite a similar use of stavudine. Possibly, the high consumption of cassava in the catchment area of our clinic known to induce endemic polyneuropathy may have increased this toxicity [47-50]. A regimen containing efavirenz and zidovudine combined with lamivudine was associated with a 2-fold higher risk of treatment modification within the first year of cART, possibly due to an enhanced effect of zidovudine on a pre-existing anemia in a hyperendemic area of malaria.

### Risk factors for poor survival

CD4 cells of less than 50 cells/μl and low body weight at starting cART reflecting late presentation were independent risk factors for poor survival in our cohort, findings shared with other cohorts [8,12,14,23,38,42,43,46,51-54]. Additionally, May et al. [55] found male sex, age > 40 years and severe anemia to be prognostic. Interestingly, active tuberculosis was not a risk factor for poor survival in our cohort, although tuberculosis has been estimated to cause about one third of all deaths of HIV-infected individuals in sub-Saharan Africa [4,43,52]. However, our study may be underpowered to detect differences in survival as only few patients had tuberculosis. Noteworthy, patients with tuberculosis were enrolled for treatment at higher CD4 cell counts. All patients had a mandatory adherence supporter, but data collection on adherence itself was inconsistent. Hence, we could not analyze adherence in this setting as in comparable African settings [14,24,25,46], where good adherence was shown to be associated with improved outcome under cART.

In our study, a considerable proportion of patients died or was lost to follow-up adding up to 21.7% during the first year of cART. Three comprehensive reviews [56-58] showed that attrition was highest during the first year on cART and 12-months' retention rate ranged between 55%-93% [56-58] with an average rate of 75.2%-77.5% [57,58], which was comparable to our evaluation. Loss to follow-up was found to be more likely in individuals with WHO stage 4, CD4 cell count less than 50 cells/μl [42,43,51] and within the first 3 months of starting cART [47,51]. We assume that a significant part of these patients have died as an evaluation by Brinkhof showed [29]. The remote rural setting with distances up to 150 kilometers from the treatment centre may have contributed to this result [23,59]. Nevertheless, the 1-year survival on cART was comparable with other studies including urban settings [8,35,42,47,51-58].

### Limitations and strengths

We acknowledge some limitations of our study. First, data on follow-up was not complete for all patients, possibly leading to an overestimation of cART effectiveness in this setting. Secondly, a significant proportion of patients were lost to follow-up (14.8%). However, as we considered that these latter patients experienced treatment failure this may have accounted for an underestimation of cART effectiveness. Thirdly, viral load could not be measured which has important implications for the definition of treatment failure. Moreover, the exact cause of death was not known in 43% of all patients. This may have lead to a survival bias as tuberculosis may have accounted for a substantial proportion of deaths. Adherence could not be included in our analysis due to inconsistency of data. Due to the large proportion of missing data for certain variables, we could not include all investigated factors in the multivariate analysis. WHO's 2010 guidelines [60] promote tenovofir as one of the first line compounds of cART, which limits the relevance of our data to a certain extent. Nevertheless, stavudine will probably remain for a while due to its low costs, wide availability and being an alternative compound in a setting with a relevant prevalence of HIV associated nephropathy [61-63] and increasing prevalence of arterial hypertension and diabetes mellitus contributing to impaired renal function. This may especially be relevant in settings with high prevalence of anemia due to malaria and nutritional deficiencies where zidovudine can't be readily used.

The strengths of our study are manifold. The care and treatment facility at St. Francis Designated District Hospital is among the largest centres offering cART in a remote rural setting of Eastern Africa. Data was collected prospectively allowing more precise assessment of treatment efficacy. Data was derived from one centre therefore avoiding any leveling effects and high heterogeneity. Also, our site strictly follows the Tanzanian guidelines of the NACP and was uniquely supplied by the NACP which directly reflects a reach-out antiretroviral treatment programme with a public health approach. All individuals in this study were treatment-naïve at the start of cART. Therefore, results are not affected by previous exposure to antiretrovirals. Finally, our cohort is one of the largest rural HIV-cohorts in sub-Saharan Africa [13,23-27] showing a 1-year survival on cART similar to studies performed in urban settings [8,35,42,46,51,52], where education is higher, infrastructures, skilled human resources, patient support and referral systems are better than in a rural setting. Starting cART with the support of a treatment assistant may have helped to overcome many of the above mentioned constraints of a rural setting [64,65]. These findings strongly support the feasibility of WHO's policy of "rolling and reaching out" [5] to rural sites in sub-Saharan Africa.

## Conclusions

In conclusion, cART initiation at higher CD4 cell counts and better general health condition can reduce HIV related mortality in a rural African setting. The principle of starting cART only with a treatment assistant may help to overcome the constraints of a rural setting and allows comparable treatment outcomes as in urban cohorts. Efforts must be made to promote earlier HIV diagnosis in order to start cART timely and improve survival in rural African regions with moderate to high HIV prevalence. This necessitates wider voluntary HIV testing and counselling.

## Competing interests

The authors declare that they have no competing interests.

## Authors' contributions

The authors designed and executed the study, had full access to the raw data, performed all analyses, wrote the article and had full responsibility for the decision to submit for publication. All authors contributed to the design of the present analysis and to the final draft of the article. The authors EM, MS, EGM and EC were members of the Chronic Disease Clinic of St. Francis Designated District Hospital at Ifakara, Kilombero District, United Republic of Tanzania and were actively involved in the management of patients and the collection of data. EM completed the first draft of the article. LE performed the statistical analyses.

## Pre-publication history

The pre-publication history for this paper can be accessed here:

http://www.biomedcentral.com/1471-2334/11/98/prepub
